# Lercanidipine’s Antioxidative Effect Prevents Noise-Induced Hearing Loss

**DOI:** 10.3390/antiox13030327

**Published:** 2024-03-07

**Authors:** Zhaoqi Guo, E Tian, Sen Chen, Jun Wang, Jingyu Chen, Weijia Kong, Debbie C. Crans, Yisheng Lu, Sulin Zhang

**Affiliations:** 1 Department of Otorhinolaryngology, Union Hospital, Tongji Medical College, Huazhong University of Science and Technology, Wuhan 430022, China; zhaoqi_guo@hust.edu.cn (Z.G.); etian@hust.edu.cn (E.T.); senchen@hust.edu.cn (S.C.); ent_wangjun@hust.edu.cn (J.W.); m202175891@hust.edu.cn (J.C.); entwjkong@hust.edu.cn (W.K.); 2 Institute of Otorhinolaryngology, Union Hospital, Tongji Medical College, Huazhong University of Science and Technology, Wuhan 430022, China; 3 Cell & Molecular Biology Program, Colorado State University, Fort Collins, CO 80523, USA; 4 Department of Physiology, School of Basic Medicine, Tongji Medical College, Huazhong University of Science and Technology, Wuhan 430030, China; 5 Institute of Brain Research, Collaborative Innovation Center for Brain Science, Huazhong University of Science and Technology, Wuhan 430030, China

**Keywords:** lercanidipine, noise-induced hearing loss, oxidative stress

## Abstract

Noise-induced hearing loss (NIHL) is a prevalent form of adult hearing impairment, characterized by oxidative damage to auditory sensory hair cells. Although certain dihydropyridines, the L-type calcium channel blockers, exhibit protective properties against such damage, the ability of third-generation dihydropryidines like lercanidipine to mitigate NIHL remains unclear.We utilized glucose oxidase (GO)-treated OC1 cell lines and cochlear explants to evaluate the protective influence of lercanidipine on hair cells. To further investigate its effectiveness, we exposed noise-stimulated mice in vivo and analyzed their hearing thresholds. Additionally, we assessed the antioxidative capabilities of lercanidipine by examining oxidation-related enzyme expression and levels of oxidative stress markers, including 3-nitrotyrosine (3NT) and 4-hydroxynonenal (4HNE). Our findings demonstrate that lercanidipine significantly reduces the adverse impacts of GO on both OC-1 cell viability (0.3 to 2.5 µM) and outer hair cell (OHC) survival in basal turn cochlear explants (7 µM). These results are associated with increased mRNA expression of antioxidant enzyme genes (*HO-1*, *SOD1/2*, and *Txnrd1*), along with decreased expression of oxidase genes (*COX-2*, *iNOS*). Crucially, lercanidipine administration prior to, and following, noise exposure effectively ameliorates NIHL, as evidenced by lowered hearing thresholds and preserved OHC populations in the basal turn, 14 days post-noise stimulation at 110 dB SPL. Moreover, our observations indicate that lercanidipine’s antioxidative action persists even three days after simultaneous drug and noise treatments, based on 3-nitrotyrosine and 4-hydroxynonenal immunostaining in the basal turn. Based on these findings, we propose that lercanidipine has the capacity to alleviate NIHL and safeguard OHC survival in the basal turn, potentially via its antioxidative mechanism. These results suggest that lercanidipine holds promise as a clinically viable option for preventing NIHL in affected individuals.

## 1. Introduction

Hearing loss affects ~5% of the population worldwide of all ages [1], substantially impacting the patient’s life quality due to the communication barrier. Noise exposure is the most common cause of hearing loss in adults [2]; intense or long-term noise exposure induces auditory sensory hair cell (HC) death, ribbon synapse loss, and spiral ganglion degeneration [3], resulting in permanent threshold shift (PTS). The key element contributing to noise-induced hearing loss (NIHL) is oxidative damage to hair cells (HC) via the accumulation of reactive oxygen (ROS) and nitrogen (RNS) species [4,5,6]. Early interventions that neutralize or eliminate free radicals can attenuate noise-induced hair cell death and NIHL occurrences [7,8,9].

Antioxidants are chemicals that impede or delay the oxidation process in low concentrations compared to the oxidizable substrates. However, the in vivo efficacy of traditional exogenous antioxidants, such as β-carotene, vitamins C and E, and N-acetyl cysteine, is becoming increasingly debatable [10,11]. Dihydropyridines, calcium channel modulators frequently administered to manage elevated blood pressure, possess antioxidant properties and are regarded as a distinct group of prospective antioxidants. A third-generation dihydropyridine, lercanidipine, surpasses previous iterations in terms of safety and effectiveness in treating cardiovascular disorders due to its combined function as a calcium channel blocker and antioxidants [12]. Lercanidipine has demonstrated its capability to suppress oxidative stress in the context of iron-mediated nephropathy and cerebral ischemia [13,14].

We speculated that lercanidipine’s antioxidant activity contributes to its protective effect against NIHL. In this work, we (1) investigated the potential effects of lercanidipine on the viability of hair cell-like OC-1 cells and HC survival rate in cochlea explants under oxidative stress; and (2) examined the oxidative stress product and HC number in the NIHL mouse model. This study provides initial validation of lercanidipine’s potential employment in treating NIHL.

## 2. Materials and Methods

### 2.1. Animals

A total of 48 C57BL/6J mice were used for cochlear explant culture and in vivo experiments. Unless otherwise stated, all mice were housed in groups, subjected to a 12 h light/dark cycle, and kept at a constant temperature (23 °C–25 °C). All experimental procedures were approved by the Committee on Animal Research of Huazhong University of Science and Technology, and all efforts were made to minimize the number of mice used and their suffering.

### 2.2. Cell Culture and Treatment

Consistent with previous studies [15,16], the HC-like OC-1 cell line, which is an immortalized cochlear sensory epithelial cell line that was derived from the organ of Corti of rats and that expresses multiple HC markers, was cultured in high-glucose DMEM (11995500, Gibco, Grand Island, NY, USA) with 5% fetal bovine serum (11054001, Gibco, Grand Island, NY, CA, USA) in 5% CO_2_ at 37 °C for 24 h. Then, OC-1 cells were treated with fresh media (control group), 10 U/L GO (GO group), and 10 U/L GO along with lercanidipine (T6874, TargetMol, Boston, MA, USA) (GO + lercanidipine group) for 24 h. Following treatments, cell viability was tested using the CCK-8 assay.

### 2.3. Cell Viability Assay

Cell Counting Kit-8 (CCK-8) viability assay (CK04, Dojindo Laboratories, Kumamoto, Japan) was performed to evaluate cell viability. About 5000 cells/well were seeded in 96-well plates. After 24 h culture in fresh media and treatment for 4 h with fresh media, GO or GO + lercanidipine, and 10% CCK-8 solution were added. The absorbance of the formazan at 450 nm was detected.

### 2.4. Culture of Cochlear Explant and DRUG Treatment

The cochlear explants were dissected and cultured, as previously reported [17,18,19]. P3 mice were sacrificed by cervical dislocation and soaked in 75% alcohol, and then the cochlear basilar membrane was carefully isolated from the cochlea in the pre-cooled sterile Hank’s balanced salt solution (H1025, Solarbio, Beijing, China). The cochlear basilar membrane was transferred onto a collagen gel matrix prepared in advance. The gel droplet was a mixture of 9 μL rat tail collagen (Type 1-4236, BD Biosciences, San Jose, CA, USA), 1 μL 10 × Basal Medium Eagle (BME; B9638, Sigma-Aldrich, Saint Louis, MO, USA), 1 μL 2% sodium carbonate (P1110, Solarbio, Beijing, China). Then, the mixture was placed on the surface of a 35 mm culture dish and allowed to gel for approximately 30 min at 37 °C. All explants for primary culture were incubated at 37 °C in an atmosphere of 5% CO_2_ in the fresh media [18]. On the second day, the explants of the cochlea were divided into three groups and treated for 8 h with fresh medium, 5 U/L GO (G3660, Sigma-Aldrich, Saint Louis, MO, USA) (GO group), and 5 U/L GO together with 0.1 mM lercanidipine (GO + lercanidipine group). Each group contains 4 mice.

### 2.5. Real-Time PCR

Total RNA was extracted from cochlea explants with RNA simple Total RNA Kit (DP419, Tiangen, Beijing China) and reverse-transcribed to cDNA by cDNA Synthesis Kits (R323-01, Vazyme, Nanjing, China), according to the manufacturer’s protocols. The qRT-PCR was performed on a LightCycler 480 RT-PCR system (Roche Diagnostics Ltd., Basel, Switzerland) with the LightCycler 480 SYBR Green I Master Mix (04887352001, Roche Diagnostics, Basel, Switzerland). The abundance of different transcripts was assessed in triplicates. The primers in Table 1 were designed for each targeted mRNA or DNA (Tsingke Biotech, Beijing, China). The mRNA expression was computed using the 2^−ΔΔCt^ method and normalized to GAPDH.

### 2.6. Noise Exposure

P35 male mice in noise group and noise + lercanidipine group were exposed to a broadband noise with a frequency spectrum from 2 to 20 kHz for 1 h at 110 dB SPL to induce severe permanent threshold shifts (PTS), including the destruction of both outer hair cells (OHCs) and inner hair cells (IHCs) [20]. Generally, mice were restrained in the center of the sound chamber by a stainless-steel wire cage, and the noise was elicited by a loudspeaker driven by a computer and a power amplifier. Sound levels were calibrated with a sound level meter (Model AWA5636-1, AIWA Technology, Hongkong, China) before and after exposure. Control mice were kept in the same chamber for 1 h without noise exposure. In the noise + lercanidipine group, animals were injected intraperitoneally with a solution of lercanidipine (6 mg/kg) 1 h before and 1, 2 and 3 days after noise exposure, respectively. Each group contained 3 mice.

### 2.7. Auditory Brainstem Response (ABR) Measurements

The auditory threshold of each group (*n* = 3) was measured by ABR at 14 days post-exposure. The detailed method of ABR measurement has been described previously [21]. Briefly, mice were anesthetized with an injection of pentobarbital sodium (20 mg/kg, i.p.) and then placed on a heating pad to maintain body temperature. The recording electrode was carefully inserted subcutaneously at the apex of the skull, while the reference electrode was positioned subcutaneously within the ear. Additionally, the grounding electrode was placed subcutaneously in the ear on the contralateral ear. Tone burst stimuli at frequencies of 8, 16, 24, 32, 40 kHz were generated, and responses were recorded by the Tucker-Davis Technologies System (RZ6, Tucker-Davis Tech., Alachua, FL, USA). A loudspeaker (MF-1, Tucker-Davis Tech., Alachua, FL, USA) connected to the system was placed 5 cm away from the tested ear. Responses were averaged 1024 times, starting at 90 dB with decreasing 10 dB steps, then narrowing to 5 dB step near the threshold. The lowest sound level that elicited a consistent wave was considered as the threshold.

### 2.8. Cochlear Tissue Preparation and Fluorescent Labeling

For the in vitro experiment, cochlear explants were fixed with 4% paraformaldehyde for 1 h at room temperature, and subsequently rinsed three times with 0.1% Tween-20 in PBS (PBST).

After the ABR test, mice were deeply anesthetized and sacrificed. The cochleae were dissected from temporal bones and fixed in 4% paraformaldehyde in 0.01 M PBS for 12 h at 4 °C. After decalcification with 10% EDTA for 48 h at 4 °C, they were transferred to PBS and the flattened cochlear preparations were carefully dissected.

The cochlear explants and flattened cochlear preparations were blocked in a solution of 5% Bovine Serum Albumin in PBST for 1 h. Subsequently, the samples were incubated with primary antibodies diluted in PBS overnight at 4 °C: polyclonal rabbit anti-myosin7a antibodies (1:300, 25-6790, Proteus Bio-Science, Waltham, MA, USA), monoclonal mouse anti-3-nitrotyrosine (3-NT) antibody (1:300, ab110282, Abcam, Cambridge, UK), monoclonal rabbit anti-4-hydroxynonenal (4-HNE) antibody (1:300, ab46545, Abcam, Cambridge, UK). The samples were washed three times with PBST and then incubated with Alexa-Fluor-594- or 647- conjugated secondary antibodies at a dilution concentration of 1:200 (ANT019, ANT020, Antgene, Wuhan, China) for 2 h at room temperature. Nuclei and F-actin staining were labeled with DAPI (ANT165, Antgene, Wuhan, China) and phalloidin (P5282; Sigma-Aldrich, Saint Louis, MO, USA) for 10 min. The samples were visualized under a laser-scanning confocal microscope (A1R SI Confocal, Nikon, Tokyo, Japan).

Quantification of the fluorescence intensity was performed in accordance with the method previously reported by Hu Yuan et al. [22]. The immunolabeling of 3-NT and 4-HNE was quantified from original confocal images, taken with equal laser and photomultiplier gain, using ImageJ 1.53t (National Institutes of Health, Bethesda, MD, USA). The cochleae from the different groups were fixed, labeled simultaneously with identical solutions, and processed in parallel. All of the preparations were counter-labeled with Alexa-Fluor-594 phalloidin (red); hair cell structure was labeled to identify the comparable parts of the OHCs in confocal images. The relative fluorescence was quantified by normalizing the ratio of average fluorescence of noise + lercanidipine OHCs to the average fluorescence of the noise OHCs.

### 2.9. Statistical Analysis

All data were analyzed using Microsoft Excel LTSC MSO (Microsoft, Redmond, WA) and GraphPad Prism 9.4.0 (GraphPad Software, Boston, MA, USA). All data were presented as mean ± standard error of the mean (SEM). Two-tailed Student’s *t*-test, one-way ANOVA followed by a Dunnett multiple comparisons or two-way ANOVA were used to analyze the data. *p* values below 0.05 were considered to be statistically significant.

## 3. Results

### 3.1. Lercanidipine Alleviates the Adverse Effect of GO on the OC-1 Cell Viability

**Figure 1:**
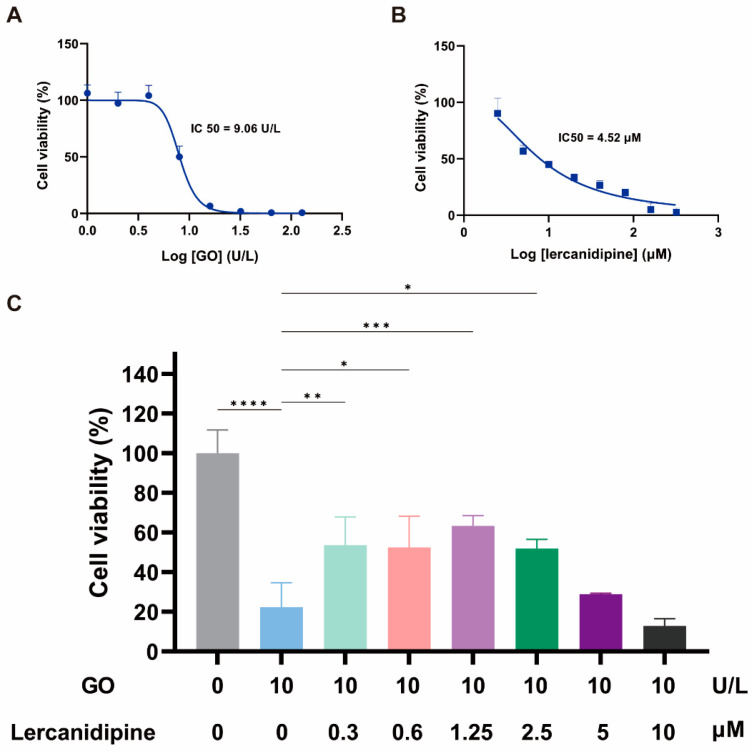
Lercanidipine alleviates the detrimental effects of GO on OC-1 cell viability. (**A**,**B**) The dose-dependence of OC-1 viability on GO and lercanidipine, where the half-maximal inhibitory concentration (IC50) was 7.5 U/L and 4.52 μM, respectively. (**C**) 0.3 to 2.5 μM lercanidipine alleviated the cytotoxity of GO treatment (24 h, 10 U/L) on OC-1 cell, while 5 to 10 μM lercanidipine did not provide any protection. (* *p* < 0.05, ** *p* < 0.01, *** *p* < 0.001, **** *p* < 0.0001. *n* = 3).

To evaluate the antioxidative effect of lercanidipine on hair cells in vitro, we first observed the concentration dependence of 24 h lercanidipine and GO treatments on hair cell-like cell line OC-1 cell viability. OC-1 cells decreased when lercanidipine and GO concentrations increased, the half inhibitory concentrations (IC) of GO and lercanidipine on OC-1 were 9.06 U/L and 4.52 μM, respectively (Figure 1A,B), and 10 U/L GO significantly reduced OC-1 cell viability to about 20%. Next, we observed the antioxidative effect of lercanidipine on OC-1 cell viability after GO + lercanidipine treatment for 24 h. Compared to the GO group, lercanidipine could significantly enhance cellular viability on 0.3 to 2.5 μM. In contrast, the high concentration of lercanidipine (5~10 μM) failed to protect OC-1 cells, possibly due to the adverse effect of the high lercanidipine concentration shown in Figure 1C.

### 3.2. Lercanidipine Reverses the Damaging Effects of GO on the Cochlear Explant

**Figure 2:**
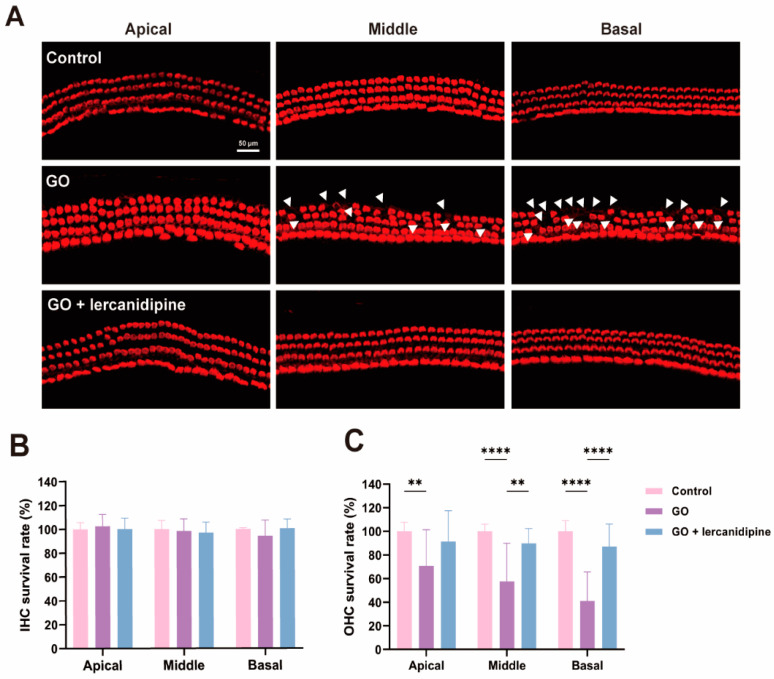
Lercanidipine reduces GO-induced OHC loss in the cochlear explant. (**A**) After 24 h culturing, cochlear explants were treated without GO, with GO or GO + lercanidipine for 8 h. Representative images of OHC and IHC from the three cochlea turns labeled with phalloidin (red). White triangles indicate cell loss. No impairment was observed in the IHC (**B**), while lercanidipine reversed the GO-induced OHC loss (**C**). (** *p* < 0.01, **** *p* < 0.0001. *n* = 4 mice).

To further confirm the antioxidative effect of lercanidipine, the cochlear explant was treated with GO or GO + lercanidipine. After 8 h GO incubation, hair cell degeneration was observed in all turns of outer hair cells (OHC), and hair cell loss in the basal turn was more severe than that in the middle and apical turns (Figure 2A,C). However, no difference was observed in the inner hair cells (IHC) (Figure 2B). Meanwhile, the survival rates of OHC were fully reversed after 8 h GO + lercanidipine (7 μM) treatment, suggesting the antioxidant efficacy of lercanidipine on hair cells (Figure 2).

### 3.3. Lercanidipine Inhibits Oxidative and Promotes Antioxidative Enzyme Gene mRNA Expression after GO Treatment of Cochlea Explant

**Figure 3:**
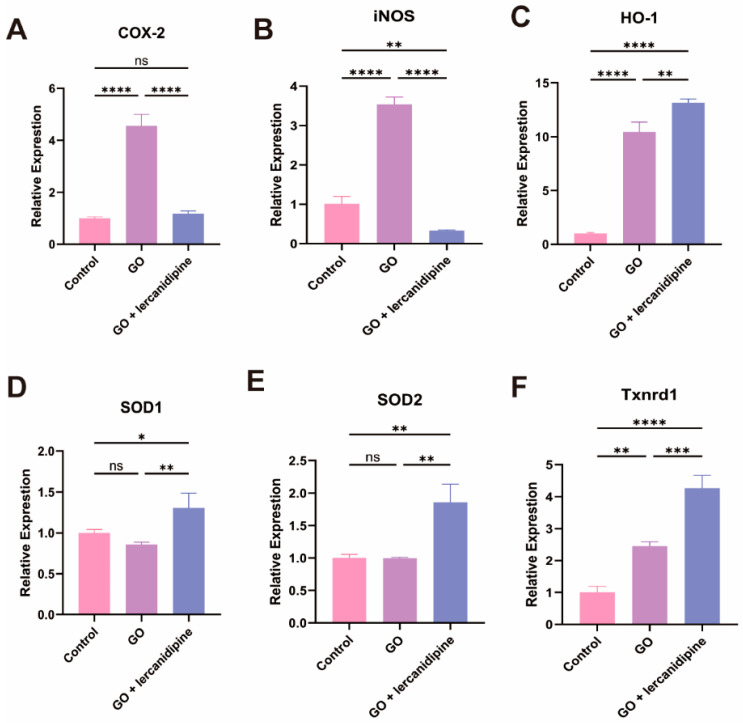
Lercanidipine inhibits oxidative and promotes antioxidative enzyme gene mRNA expression after GO treatment of cochlea explant. The mRNA levels of oxidative-related genes, Cox-2 and iNOS increased in the GO group, which could be reversed by lercanidipine (**A**,**B**). The mRNA levels of antioxidative-related genes, HO-1, SOD1, SOD2, and Txnrd1, increased in the GO + lercanidipine group, compared to the GO group (**C**–**F**). (* *p* < 0.05, ns: no significance, ** *p* < 0.01, *** *p* < 0.001, **** *p* < 0.0001. *n* = 3).

To elucidate the mechanism of lercanidipine’s antioxidative effect, the mRNA expressions of oxidation-related genes were quantified. The transcription of two oxidase genes, Cox-2 and iNOS, and two antioxidant enzyme genes, HO-1 and Txnrd1, were increased after the GO treatment. Nevertheless, in the GO + lercanidipine treatment group, the mRNA expressions of COX-2 and iNOS genes were significantly decreased (Figure 3A,B); in contrast, the expressions of antioxidant enzyme genes, HO-1, SOD-1/2, and Txnrd-1 were much higher compared to the GO group (Figure 3C–F). These results suggest that lercanidipine protects OHC, possibly via antioxidant effects.

### 3.4. Lercanidipine Attenuates NIHL and Hair Cell Loss

**Figure 4:**
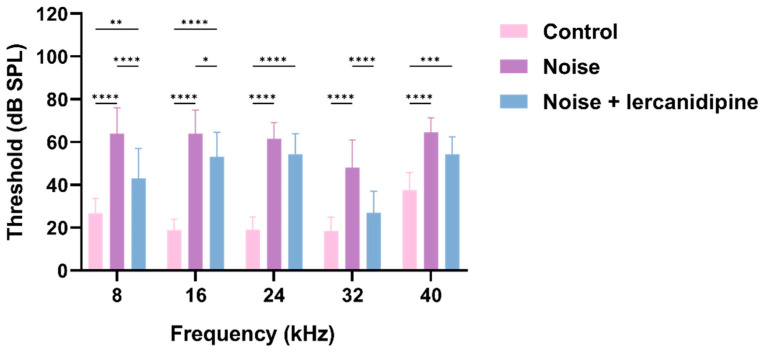
Lercanidipine attenuates noise-induced hearing loss. Fourteen days after one-hour noise exposure, the hearing threshold in the noise group was elevated, which was partially reversed by lercanidipine treatment (i.p. injection, 6 mg/kg, once before noise and once a day in post-noise 1, 2, 3 days). (* *p* < 0.05, ** *p* < 0.01, *** *p* < 0.001, **** *p* < 0.0001. *n* = 3).

**Figure 5:**
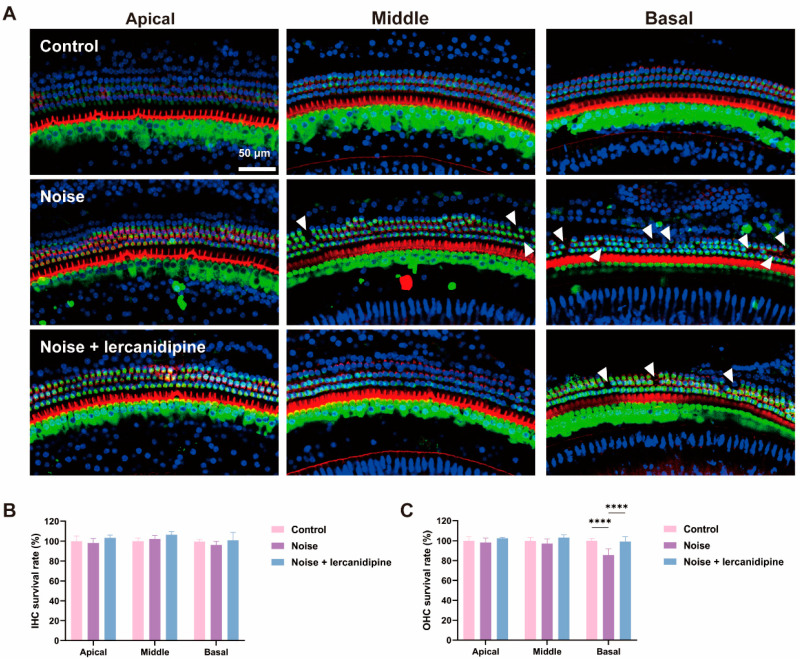
Lercanidipine attenuates noise-induced hair cell loss in OHC. (**A**) Representative images showing OHC from the three turns of the cochlea labeled with phalloidin (red) and myosin 7a (green) 14 days after 1 h noise exposure with (noise + lercanidipine group) or without (noise group) lercanidipine i.p. injection (6 mg/kg) 1 h in advance. Arrowheads indicate the missing hair cells in three turns. (**B**) No difference was observed in the IHC survival rate 14 days after noise exposure. (**C**) Lercanidipine reversed noise-induced OHC loss in the basal turn. (**** *p* < 0.0001. *n* = 3).

To investigate whether lercanidipine can attenuate NIHL, mice were treated with lercanidipine for 1 h, and then exposed to 110 dB broadband noise for 1 h. 14 days after noise exposure, the hearing threshold was elevated to about 40–50 dB, while lercanidipine significantly attenuated the auditory threshold elevation at 8, 16, 32 kHz (Figure 4). In terms of morphology, lercanidipine also protected the integrity of the cochlear sensory epithelium (Figure 5A). Moreover, quantification of OHC showed that treatment with lercanidipine reduced OHC loss in the basal turn induced by noise (Figure 5C). No significant change was observed in IHC survival rate after noise or noise + lercanidipine treatments. These results suggest that lercanidipine may alleviate NIHL via protection of OHC.

### 3.5. Lercanidipine Reduces Noise-Induced Oxidative Stress in OHCs 

**Figure 6:**
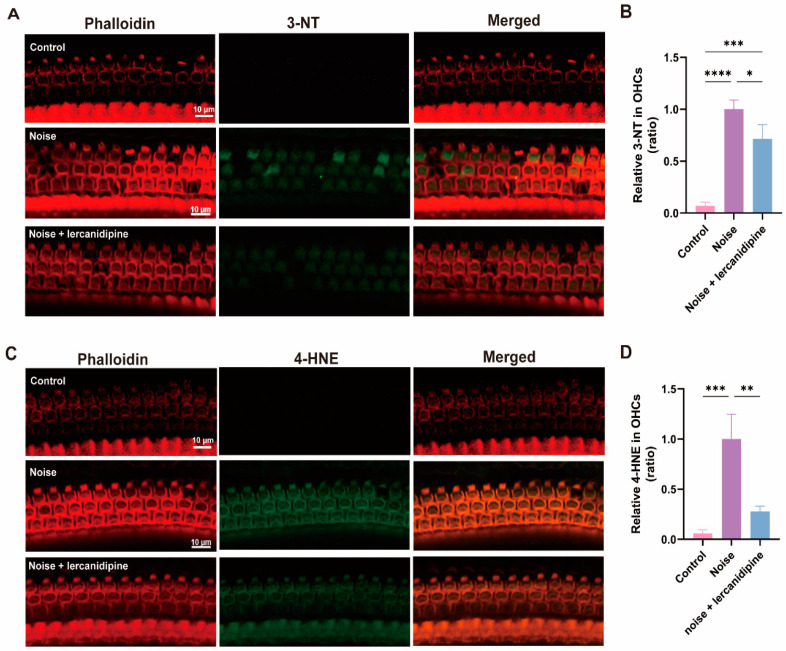
Lercanidipine reduces oxidative stress in the OHC induced by noise. 3-NT (**A**) or 4-HNE (**C**) were coimmunostained with phalloidin in the basal turn OHCs 3 d after noise exposure with (treatment group, once before noise and once a day in post-noise 1, 2, 3 days) or without (noise group) i.p. lercanidipine injection (6 mg/kg). Quantification of 3-NT (**B**) and 4-HNE (**D**) fluorescent intensity in OHCs showed a significant decrease in the noise + lercanidipine groups. (* *p* < 0.05, ** *p* < 0.01, *** *p* < 0.001, **** *p* < 0.0001. *n* = 3 mice).

To determine the effect of lercanidipine on noise-induced oxidative stress in OHCs, 3-NT and 4-HNE levels in OHCs were assessed. Treatment with lercanidipine markedly decreased 3-NT and 4-HNE immunolabeling in OHCs 3 d after exposure (Figure 6).

## 4. Discussion

This study provides evidence of lercanidipine’s efficacy in preventing NIHL, which is likely due to its antioxidant effect on OHCs. Firstly, we utilized GO to create an oxidative stress model using the OC-1 cell line and cochlear preparations. We discovered that lercanidipine, an L-type voltage-gated calcium channel blocker, could directly preserve OC-1 cells and cochlear explants from GO-induced cell loss, potentially due to lercanidipine’s antioxidant capabilities, as evidenced by the altered expression of oxidative-related genes. Additionally, we found that lercanidipine could reduce the degree of hearing loss and OHC loss caused by noise exposure, primarily through an antioxidative process, as demonstrated by the increase in 3-NT and 4-HNE fluorescence. Notably, OHCs were found to be more sensitive than IHCs, consistent with our cochlear preparation data.

Oxidative stress has been widely recognized as a major contributor to cochlear noise-induced damage [5,23,24]. Thus, we initially created an oxidative stress model using the OC-1 cell line and cochlear preparations. Similar to H_2_O_2_, GO exposure caused damage, with outer hair cells being more susceptible than inner hair cells and the basal turn being more peroneal to the apex turn [25,26].

Further analysis substantiated that lercanidipine can ameliorate NIHL through an antioxidant mechanism. In this experiment, noise exposure at 110 dB SPL for 1 h resulted in a permanent hearing threshold shift in C57 mice. The ABR results indicated that the threshold shift was slightly more profound at lower frequencies than at higher frequencies, whereas outer hair cell loss was more prominent at higher frequencies. While this seems paradoxical, it is plausible and consistent with previous reports. Shi-Nae Park et al. [3] documented that 22 days post noise exposure, the hearing threshold of C57 mice at 8 kHz was marginally higher than that at 32 kHz; however, morphological degeneration was more severe at the basal turn. Hence, C57 mice exhibited diverse ‘‘site’’ vulnerabilities. This can be rationalized by the innate apical-to-basal gradient of decreasing SOD2 expression in mammals [27]. Consequently, when noise exposure induced ROS overload, the basal turn was more vulnerable than the apex turn, thus accounting for greater OHC loss in the former than the latter. Apart from hair cell demise, the primary pathological hallmarks of NIHL comprise ribbon synapse loss, spiral ganglion degeneration, fusion, or alteration of stereocilia and supporting cell trauma [28]. There were reportedly notable reductions in outer hair cell functioning following noise exposure, which seemed more detrimental at lower frequencies compared to higher frequencies [3]. As such, it is suggested that the functional deficit of OHC at the apex might explain the heightened threshold shift at lower frequencies. 

Following noise exposure, mitochondrial metabolic activity increases, producing large quantities of ROS that are not adequately neutralized [5,23,24]. ROS migrate into the cytoplasm, augmenting superoxide and lipid peroxidation production, culminating in hair cell death through either apoptosis or necrosis. Meantime, noise and vasoactive lipid peroxidation products contribute to ischemia, followed by reperfusion, thereby exacerbating the generation of ROS [29,30,31]. Additionally, ROS can induce the synthesis of proinflammatory cytokines, further aggravating the ensuing damage [32,33]. In our study, lercanidipine mitigated the auditory threshold decline, OHC loss, and generation of lipid and protein peroxidation products (4-HNE, 3-NT) induced by noise exposure. Nonetheless, it remains uncertain whether lercanidipine directly reduces oxidation in cochlear hair cells or indirectly through other tissues. By examining the antioxidant effects of lercanidipine on cochlear explants and OC-1 cell lines, we deduced that lercanidipine directly safeguards HC loss by diminishing oxidative stress. This work represents the inaugural exploration of the protective role of lercanidipine on NIHL. Lercanidipine possessed antioxidant properties independent of calcium channel modulation, attributable to its high lipophilicity, coupled with a chemical composition that facilitates proton-donating and resonance-stabilization mechanisms, ultimately terminating the free radical chain reaction [34,35,36]. Several L-type calcium channel blockers have been previously shown to offer protection against NIHL; nevertheless, the underlying processes differ among these compounds. Verapamil and nilvadipine alleviate the transient threshold shift by curtailing vascular permeability and aggregation of platelets [37,38]. Meanwhile, nifedipine dampens the noise-induced Ca^2+^ load of hair cells and simultaneously minimizes the cochlea’s sensitivity to noise stimuli, thereby augmenting cochlear tolerance to noise, and parsimoniously decreasing noise-instigated injury to cochlear function [39]. Nevertheless, nimodipine and nifedipine did not decelerate ROS production; moreover, verapamil’s capability for antioxidant stress was inferior to that of lercanidipine [40]. 

Antioxidants have traditionally constituted one of the principal pharmaceutical approaches employed for the prevention or treatment of NIHL, including agents such as N-Acetylcysteine [9,41], glutathione, D-methionine [7,8]. Despite their efficiency in interacting with ROS, antioxidants often present limitations such as poor tissue selectivity, brief half-life, and elevated effective concentrations. In contrast, lercanidipine boasts advantages including a lengthy half-life (approximately 8–10 h), high lipophilicity, and extended duration of action. Furthermore, lercanidipine has been demonstrated to possess anti-inflammatory properties [36]. Prior research has established that lercanidipine attenuates vascular inflammation by impeding the activation of MAPK, Akt/IκB-β, and NF-кB signaling cascades, while also down-modulating MMP-2/MMP-9 and HMGB1 expression, thereby reducing the synthesis of NO, TNF-alpha, and ROS [42,43]. Given these observations, in addition to its antioxidant properties, lercanidipine may also play a crucial role in managing Ca^2+^ overload and inflammation to counteract noise-induced damage. This offers an exciting avenue for future research endeavors.

**Figure 7:**
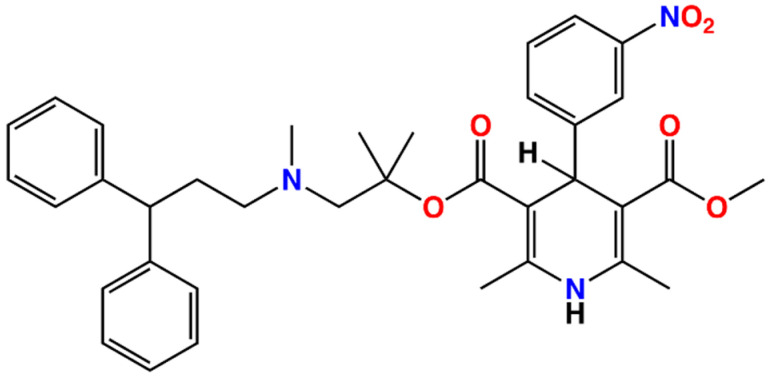
Chemical structures of the lercanidipine.

The clinical translation of lercanidipine’s impact on NIHL holds promise. Lercanidipine is a highly lipophilic third-generation dihydro-pyridine that has been extensively used for over two decades in the management of hypertension [44]. This drug demonstrates superior tolerability compared to other dihydropyridines. Daily clinical use of lercanidipine reveals an overall prevalence of adverse reactions of 6.5%, the majority of which include headaches (2.9%), ankle edema (1.2%), flushing (1.1%), and palpitations (0.6%) [45]. According to V Barrios et al. [46], the occurrence and severity of these side effects are significantly lower for lercanidipine in comparison to amlodipine/nifedipine. Chemically, lercanidipine differs structurally from other dihydropyridines due to the presence of an amine group, rendering it positively charged in blood plasma, consequently displaying a distinct distribution pattern relative to the neutral dihydropyridines. Furthermore, its hydrophobic nature distinguishes it from other dihydropyridines, possibly making it more easily absorbed and capable of traversing membranes (Figure 7). Future studies could capitalize on these fundamentally unique properties to investigate whether the combination of lercanidipine, glucocorticoids, N-acetylcysteine, or neurotrophic factors confers enhanced hearing preservation in individuals suffering from hypertension compared to monotherapy. Given that oxidative stress serves as the primary conduit for sudden hearing loss and age-associated hearing loss [47,48,49,50], it would be apt to conduct cellular and animal trials in the future. If lercanidipine proves efficacious, it would be particularly suited for individuals with hypertension and NIHL, reducing the burden of therapy required.

## 5. Conclusions

These results suggest that lercanidipine alleviates NIHL by promoting HC survival via its antioxidative effect. Lercanidipine is already a widely used and well-tolerated clinical drug; therefore, it is very promising to investigate the effect of lercanidipine on treating human NIHL. 

## Figures and Tables

**Table 1:** The primers designed for real-time PCR.

Gene ID	Gene	Gene Location	Amplicon Size	Sequence (5′→3′)	CDS Location	Exon
19225	COX-2	NC_000067.7 (149975851..149983978)	271	TTCAACACACTCTATCACTGGC AGAAGCGTTTGCGGTACTCAT	1099–1120 1369–1349	8–9
18126	iNOS	NC_000077.7 (78811613..78851052)	127	GTTCTCAGCCCAACAATACAAGA GTGGACGGGTCGATGTCAC	103–125 229–211	1–3
15368	HO-1	NC_000074.7 (75820246..75827221)	100	AAGCCGAGAATGCTGAGTTCA GCCGTGTAGATATGGTACAAGGA	80–100 179–157	2
20655	SOD1	NC_000082.7 (90017650..90023221)	139	AACCAGTTGTGTTGTCAGGAC CCACCATGTTTCTTAGAGTGAGG	83–103 221–199	2–3
20656	SOD2	NC_000082.7 (90017650..90023221)	113	CAGACCTGCCTTACGACTATGG CTCGGTGGCGTTGAGATTGTT	86–107 198–178	2
50493	Txnrd1	NC_000076.7 (82669785..82733558)	134	CCCACTTGCCCCAACTGTT GGGAGTGTCTTGGAGGGAC	76–94 209–191	1

## Data Availability

The data presented in this study are available on request from the corresponding author.
